# Adolescents’ Mental Health at School: The Mediating Role of Life Satisfaction

**DOI:** 10.3389/fpsyg.2021.720628

**Published:** 2021-08-18

**Authors:** Valeria Cavioni, Ilaria Grazzani, Veronica Ornaghi, Alessia Agliati, Alessandro Pepe

**Affiliations:** Lab for Developmental and Educational Studies in Psychology, “R. Massa” Department of Human Sciences for Education, University of Milano-Bicocca, Milan, Italy

**Keywords:** school mental health, adolescence, life satisfaction, teacher-student relationship, school connectedness, structural equation modelling

## Abstract

In this study, we further developed prior research on risk and protective factors in adolescents’ mental health. More specifically, we used structural equation modelling to assess whether relationships at school with teachers and peers, and life satisfaction predicted mental health in a large sample of adolescents, while also testing for age and gender invariance. The sample comprised 3,895 adolescents (*M*_age_ = 16.7, *SD* = 1.5, 41.3% girls), who completed self-report instruments assessing their perceived life satisfaction, student-teacher relationship, school connectedness and mental health. Overall, the results suggested that life satisfaction acted as a mediator between adolescents’ positive school relations and their mental health. Outcomes were invariant across genders, while quality of school relations and mental health declined with age. Limitations of the study and futures lines in mental health research among adolescents are briefly discussed.

## Introduction

According to the World Health Organization, mental health is ‘*a state of well-being in which every individual realizes his or her own potential, copes with the normal stresses of life, works productively and fruitfully, and is able to make a contribution to her or his community*’ (World Health Organization, 2005, p. 12). This definition recognises mental health as a dimension of overall health that spans a continuum from high-level wellness to severe illness, emphasising the key role of positive feelings, a sense of mastery and positive functioning (Galderisi et al., 2015).

Adolescence is widely known to be a sensitive period of exposure to a range of mental disorders whose incidence has been increasing in recent decades (Patton et al., 2014; Erskine et al., 2015). Indeed, approximately 20% of school students are now affected by diagnosable mental illnesses, with half of all mental issues developing by 14 years (Ford et al., 2003; Gore et al., 2011). Data from studies with adolescents indicate that anxiety, depression, eating disorders, bipolar disorder, personality disorders, psychosis, addictive disorders, substance abuse, suicide attempts and self-harm are all becoming more frequent among this demographic (Paus et al., 2008; Twenge et al., 2018; Burstein et al., 2019). In most cases, disorders of this kind remain undetected and, consequently, untreated until later in life (Kessler et al., 2007; Patel et al., 2007). Furthermore, depression in young people is a highly prevalent illness worldwide, and suicide is the third most frequent cause of death among adolescents in the United States and Europe (World Health Organization, 2012; Keyes et al., 2019; Twenge, 2020).

The literature shows that adolescent mental health is influenced by both individual attributes and the everyday life contexts where adolescents grow up, including school, which is a key developmental setting (Weare and Nind, 2011; Cefai and Cavioni, 2015). Students who experience mental health difficulties at school tend to exhibit poor school adjustment, reduced concentration, low achievement, problematic social relationships and a higher rate of health risk behaviours, such as substance use, school dropout and incurring expulsion (Valdez et al., 2011; Suldo et al., 2014; Farina et al., 2021).

Recent research also suggests that older adolescents may suffer a decline in mental health outcomes, with older girls reporting poorer mental health than older boys (Inchley et al., 2020). This evidence has led to growing awareness of the need to address adolescents’ mental health requirements by identifying the factors that can promote or hinder their mental health in the school setting (Eccles and Roeser, 2011; Cavioni et al., 2020a). Accordingly, the aim of this study was to investigate the role of life satisfaction and school relations, as protective and risk factors for mental health, in a large sample of adolescents.

## Protective and Risk Factors Associated with School Mental Health in Adolescence

Protective factors for mental health may be defined as individual and environmental characteristics that foster healthy development but also reduce the negative impact of risk factors for mental health issues (Deković, 1999; O’Connell et al., 2009). In contrast, mental health risk factors can increase a person’s chances of developing psychological illness (World Health Organization, 2012). In this research, we examined three factors that impact on adolescents’ mental health. The first is an individual factor, life satisfaction, while the second and third are school contextual factors, namely, student-teacher relationship and sense of community at school. Indeed, the literature on adolescent mental health is notably lacking in research on how the contextual factors associated with quality of school relationships interact with individual characteristics, such as life satisfaction, to shape mental health outcomes (Torsheim and Wold, 2001; Craig et al., 2020). In the next section, we review the existing research findings for each of these key factors.

### Individual Factor: Life Satisfaction

Life satisfaction is defined as ‘the global assessment of a person’s quality of life according to his own chosen criteria’ (Andrews and Withey, 1976, p. 478). Numerous studies have found that adolescents with higher levels of life satisfaction display better school adaptive functioning, in terms of self-efficacy, self-esteem, engagement, academic achievement, social acceptance and peer relationships, as well as lower levels of school absenteeism, dropout and behavioural problems (Proctor et al., 2009; Fergusson et al., 2015). Conversely, adolescents with low life satisfaction are more likely to display internalising and externalising problems, social stress and substance abuse (Zullig et al., 2001; Haranin et al., 2007).

### Contextual Factors: Student-Teacher Relationship and Sense of Community at School

At the contextual level, multiple school-related factors have been associated with positive mental health outcomes in adolescents. More specifically, student-teacher relationship and sense of school community are known to serve as key protective factors *via* the provision of emotional and social support and safe environments (Eccles and Roeser, 2011). Overall, such contextual factors are associated with a lower likelihood of negative mental health outcomes, such as perceived stress, health complaints and unhealthy behavior (Lemma et al., 2014). In turn, adolescents with better mental health tend to display higher levels of attainment, decision-making ability, problem-solving skills and academic achievement (Bonell et al., 2014; Suldo et al., 2014).

With regard to the student-teacher relationship, past research has generally assessed its positive and negative aspects and their implications for a wide range of student mental health outcomes. For example, Murray and Greenberg (2001) proposed that the relationship is formed of four components. Two concern the positive aspects of the relationship, which are affiliation with the teacher and a sense of bonding with school, and two its negative characteristics, which are dissatisfaction with the teacher and perceived school dangerousness. Studies on the positive aspects of the student-teacher relationship have shown that when it is characterised by warmth, respect, support and openness, it can have positive effects on students’ attitudes towards school, fostering an increased sense of belonging and more effective learning (Doll et al., 2004; Suldo et al., 2009). Pianta and colleagues have extensively documented the relationship between higher levels of attachment to teachers and bonding with the school and enhanced social and emotional skills, school satisfaction, engagement and motivation on the part of the students (e.g. Pianta, 1999; Pianta et al., 2003; Pianta and Hamre, 2009). During adolescence, affiliation with teachers and bonds with the school can shield young people from the effects of stressful life events, promoting resilience and decreasing the likelihood of developing mental health issues, such as depression and misconduct (Murray and Greenberg, 2001; Wang et al., 2013; Lemma et al., 2014; Cefai et al., 2015, 2018; Höltge et al., 2021). In contrast, dissatisfaction with teachers and perceived school dangerousness bear the potential to negatively affect mental health outcomes (Mameli et al., 2018). Indeed, studies with adolescents suggest that higher levels of dissatisfaction with teachers are associated with poor social functioning and increased peer problems, anxiety and somatic symptoms (Mameli et al., 2018), whereas perceptions of poor school safety are linked with internalising and externalising behavior problems and bullying (Nijs et al., 2014).

The sense of community in a school, also termed ‘school connectedness’ or ‘belongingness in the school’, is the other contextual factor that we view as a significant marker for mental health. It may be defined as ‘the extent to which students feel personally accepted, respected, included, and supported by others in the school social environment’ (Goodenow, 1993, p. 80). Numerous scholars have examined the impact of sense of community on adolescent mental health, finding that school connectedness is positively associated with healthy behaviours, school engagement and adjustment, motivation, school attendance, conflict resolution skills and prosocial behavior (Vieno et al., 2005; Center for Disease Control and Prevention, 2009). Conversely, sense of community is negatively associated with school absenteeism, loneliness, worry, social isolation, emotional distress, antisocial and risky behaviours, violence, delinquency, suicidal ideation and suicide attempts (Hirschi, 1969; Pretty et al., 1994; Joyce and Early, 2014).

### Age and Gender Differences in Mental Health Outcomes

Cohort studies with adolescent samples indicate that the incidence of mental health disorders increases with age, especially emotional and conduct disorders, and psychosomatic problems (Bell et al., 2019). According to the recent Health Behavior in School-aged Children report, adolescents’ quality of school relationships and school belonging worsen over time. In contrast, younger adolescents report higher levels of life satisfaction and better mental health (Inchley et al., 2020).

With respect to gender, research has long shown that adolescent males and females tend to display different mental health patterns in relation to internalising and externalising behavioural problems (Yeo et al., 2007; Nijs et al., 2014). For example, various studies have found that girls display more frequent and intense internalising behaviours, such as stress and anxiety (e.g. concerning interpersonal relationships, school demands, family relationships, and personal and social adjustment), as well as a higher risk of developing depression compared to males (Angold and Rutter, 1992; Compas et al., 1993; Nolen-Hoeksema and Girgus, 1994). On the contrary, male adolescents are more prone to externalising behaviours, such as school problems, aggressive behavior and difficulty in managing negative emotions (Stark et al., 1989; van der Ende and Verhulst, 2005).

## Research Aims and Hypothesis

Although the existing research offers insights into the role played by individual and contextual factors in secondary school students’ mental health, most previous studies have taken a deficit-based approach by focusing on the impact of negative factors (Ben-Arieh, 2000; Antaramian et al., 2010). Consequently, a number of authors have pointed out the need to focus on the role of both risk and protective factors and how they simultaneously impact on mental health (e.g. Seligman and Csikszentmihalyi, 2000; Terjesen et al., 2004). Furthermore, despite growing awareness of the importance of relational experience at school, relatively few studies have focused on the quality of students’ social relationships with teachers and peers and its impact on overall mental health during adolescence (Torsheim and Wold, 2001; Gaete et al., 2016).

**Figure 1:**
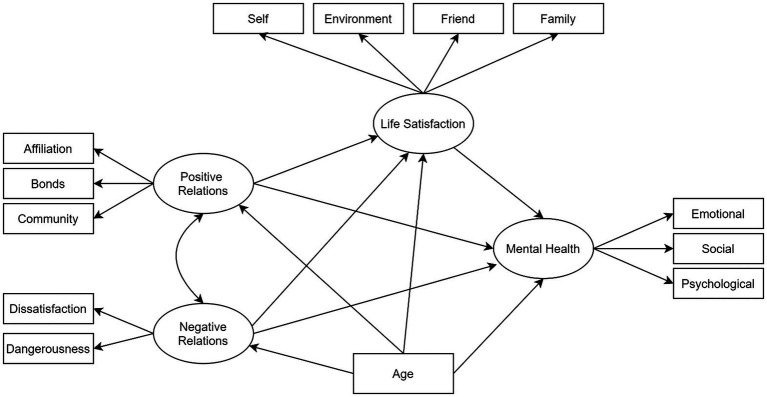
Conceptual model of association between type of relationships at school, life satisfaction and mental health in adolescents.

To address this gap, we set out to extend the recent literature on school-related factors in adolescents’ mental health by focusing on both protective and risk factors. More specifically, we used structural equation modelling (SEM) to evaluate the contributions of quality of school relations and life satisfaction to the mental health of a large group of adolescents. The model was also specified to control for effects of age, whereas a multigroup invariance test was conducted to assess differences as a function of gender. The conceptual model is illustrated in Figure 1.

Specifically, we expected that positive relations at school, in terms of affiliation with teachers, bonds with school and sense of school community, would be positively associated with both life satisfaction and mental health (Hypothesis 1); moreover, we predicted that negative relationships, in terms of dissatisfaction with teachers and a perception of school dangerousness, would be negatively associated with both life satisfaction and mental health (Hypothesis 2). In addition, we planned to conduct a multigroup invariance test to assess whether were differences between boys and girls in terms of the magnitude of the predicted associations. Notably, while past studies have examined gender differences in relation to life satisfaction and mental health, little research to date has attempted to model these variables in relation to the quality of young people’s relationships at school.

## Materials and Methods

### Participants and Procedures

The head teachers of 20 high schools located in two regions of Northern Italy (Lombardy and Piedmont) were contacted *via* email or by telephone and informed about the aims of the study and the research procedure. Seventeen schools agreed to participate in the study. A briefing letter was sent to the students’ parents. Informed consent and GDPR consent were obtained from all respondents and from their parents (who were free to deny their child’s participation in the study). Data were collected anonymously, and students were free to withdraw at any time during the administration of the questionnaire. Seventeen parents refused permission for their child to participate in the study; 11 students were absent during data collection, and four declined to participate.

Students responded to a set of online questionnaires in the classroom during regular school hours. The entire battery of instruments took about 15 min to complete. The questionnaires were presented in random order to minimise potential sources of measurement error (Franke, 1997). Data collection was managed by the lead researcher and two research assistants, who presented the aims of the study to the students before administering the questionnaires.

A sample of 3,895 students (41.3% girls) completed the research instruments. Following a review of missing data, no questionnaires were removed from the analysis due to the use of mandatory fields in the online data collection. The final sample, aged between 15 and 19 years (*M* = 16.7, *SD* = 1.5), included students from all three branches of the Italian high school system: professional institutes, technical institutes and lyceums. Almost half the participants (48%) were enrolled at lyceums (academic track), compared to 25.1 and 26.9% who attended professional and technical schools (vocational track), respectively. The majority (88.7%) were Italian citizens (i.e., born in Italy of Italian parents), while 11.3% came from non-Italian ethno-cultural backgrounds (parents not Italian and/or not born in Italy). The study was approved by the Ethical Committee of Milano-Bicocca University and was conducted in accordance with the Declaration of Helsinki.

### Measures

#### Demographics

Participants were asked to specify their age, gender, school grade and nationality.

#### Life Satisfaction

The Italian translation of the abbreviated Multidimensional Students’ Life Satisfaction Scale (MSLSS) was used to assess life satisfaction (Huebner, 1994; Huebner et al., 1998; Zappulla et al., 2014). The MSLSS is a thirty-item self-report questionnaire that measures children’s and adolescents’ life satisfaction in five domains: family (e.g. ‘I enjoy being at home with my family’), friends (e.g. ‘My friends treat me well’), school (e.g. ‘I look forward to going to school’), self (e.g. ‘Most people like me’) and living environment (‘My family’s house is nice’). Respondents are asked to rate items on a 6-point Likert scale, ranging from 1 (strongly disagree) to 6 (strongly agree). Previous studies with adolescent samples yielded acceptable validity and reliability coefficients across the five domains with alphas ranging from 0.71 to 0.91 (Gilligan and Huebner, 2007; Sawatzky et al., 2009; Huebner et al., 2012; Zappulla et al., 2014). Cronbach’s reliability values for the present study were as follows: family (*α* = 0.89), friend (*α* = 0.85), environment (*α* = 0.75), self (*α* = 0.73) and school (*α* = 0.79).

#### Student-Teacher Relationship

Students’ perceptions of their relationships with teachers and bonds with school were evaluated using the Italian version of the Student-Teacher Relationship Questionnaire (STRQ; Tonci et al., 2012) developed by Murray and Greenberg (2001). The STRQ comprises 22 items to be rated on a 4-point Likert scale (from 1 = almost never or never true to 4 = almost always or always true). The questionnaire evaluates the quality of individual students’ relationships with their teachers and their perceptions of their school environment in terms of four factors: affiliation with teacher (e.g. ‘My teachers pay a lot of attention to me’), dissatisfaction with teachers (e.g. ‘I feel angry with my teacher’), bonds with school (e.g. ‘I feel safe at my school’) and school dangerousness (e.g. ‘My school is a dangerous place to be’). The four factors have been found to be reliable, with Cronbach’s alphas ranging between 0.66 and 0.88 (Murray and Greenberg, 2001). Cronbach’s reliability values in the present study were as follows: affiliation (*α* = 0.84), bonds (*α* = 0.71), dangerousness (*α* = 0.66) and dissatisfaction (*α* = 0.74).

#### Sense of Community in School

Students’ sense of community was assessed *via* the Italian version of the Students’ Sense of Community in School scale (Vieno et al., 2005), originally developed by Samdal et al. (1998). The questionnaire comprises six items (e.g. ‘I feel I belong at this school’) designed to assess the three dimensions of sense of community (membership, shared emotional connection and fulfilment of needs) identified by McMillan and Chavis (1986). Responses are rated on a 5-point Likert scale (from 1 = strongly disagree to 5 = strongly agree). In previous studies, this questionnaire displayed satisfactory internal reliability with alphas ranging between 0.71 and 0.82 (Vieno et al., 2005; Prati et al., 2020). Cronbach’s reliability value for the present study was *α*=0.75.

#### Mental Health

The Italian validated version of the Mental Health Continuum Short Form (MHC-SF) was used to assess respondent’ mental health (Petrillo et al., 2015). This instrument, originally developed by Keyes (2002), is a self-report questionnaire comprising 14 items. Informed by Keyes’ theoretical model of mental health (Keyes, 1998, 2002, 2005, 2007; Keyes and Waterman, 2003), the MHC-SF evaluates three dimensions of mental health: emotional (e.g. ‘How often did you feel happy?’), social (e.g. ‘How often did you feel that you had something important to contribute to society?’) and psychological (e.g. ‘How often did you feel good at managing the responsibilities of your daily life?’). Items are rated on a 6-point Likert scale ranging from 0 (none of the time) to 5 (all of the time), based on respondents’ experience over the preceding month. Total scores on the MHC-SF range from 0 to 70, with higher scores reflecting better mental health. Among adolescents, the MHC-SF has displayed validity and satisfactory internal reliability values ranging from 0.75 to 0.91 (Lim, 2014; Petrillo et al., 2014, 2015; Luijten et al., 2019; Reinhardt et al., 2020). Cronbach’s reliability values for the present study were as follows: emotional (*α* = 0.81), social (*α* = 0.78) and psychological (*α* = 0.83).

### Statistical Analysis and SEM

In order to test the network of associations between adolescents’ mental health and the other study variables, we adopted a SEM approach (Thakkar, 2020). SEM techniques are based on multivariate data analysis and combine empirical measurement with theoretical inquiry by allowing latent factors to be estimated along with patterns of associations among observed variables (Guo et al., 2009; Cavioni et al., 2020b; Farina et al., 2020; Veronese et al., 2020). SEM permits estimation of the magnitude and direction of paths among variables and the evaluation of total, direct and indirect effects (Hair et al., 2017).

It is essentially a method of testing hypotheses *via* a confirmatory rather than an exploratory approach. In the present paper, we estimated a model with four latent variables and 13 empirical indicators (see Figure 1).

Moving from left to right in the figure, two of the latent constructs were *positive* and *negative school relationships* as operationalised by five empirical indicators: affiliation with teachers, bonds with school, sense of community, dissatisfaction with school and school dangerousness. Next, *life satisfaction* was assessed in relation to four domains: self, friends, family and environment. The school domain of the MSLSS was omitted from the analysis due to its collinearity with other latent variables (e.g. *positive* and *negative school relations*). Finally, the target latent variable *mental health* was modelled *via* its psychological, emotional and social dimensions. In line with the research hypotheses, we estimated the direct effects of *positive and negative school relations* on both *life satisfaction* and *mental health.* All variables were viewed as endogenous conceptual components of the model, whereas participants’ age was modelled as an exogenous variable. Estimated total effects were broken down into direct and indirect effects (Kline, 2015).

Following standard procedures for SEM, we evaluated the following goodness-of-fit indices: root mean square error of approximation (RMSEA; RMSEA < 0.05; Madeu-Olivares et al., 2018), standardised root mean square residual (SRMR; SRMR < 0.05) (Sass et al., 2014); normed fit index (NFI; NFI > 0.95; Marsh et al., 2013), Tucker-Lewis index (TLI; TLI > 0.95; Morin et al., 2013) and comparative fit index (CFI; CFI > 0.95; Morin et al., 2013). As currently recommended (e.g. Bollen and Long, 1993; Kaplan and Depaoli, 2012) for SEM, we used both Monte Carlo simulation and bootstrapping methods to estimate confidence limits with a set of random samples (*k* = 500). We calculated the given indirect effects for each of the k samples and the mean value for the selected pool of samples. Finally, we conducted a multigroup invariance test (MGCFA) to determine whether similar response patterns were obtained across gender-based cohorts. The MGCFA also helped us to specify the model structure with the best potential for generalisation (Brown et al., 2017). The hypothesis of measurement invariance was to be accepted if configural invariance (all parameters free to vary but structural model held constant), metric invariance (factor loadings set to be equal in both groups), scalar invariance (factor loadings and item intercepts constrained) and full invariance (all parameters were equivalent across the groups) were all supported. Structural equivalence was to be rejected if the indexed variations were statistically significant. The cut-off points (CFI, RMSEA and SRMR) for rejecting measurement invariance were set at Δ = 0.01, corresponding to a p level of 0.01 (Chen, 2007).

We also estimated Mahalanobis’ distance (*p* < 0.001) to detect potentially multivariate outliers; no cases needed to be removed from the dataset. Finally, we assessed the distribution of the data for each of the study measures. None of the kurtosis or skewness values exceeded the recommended limits [−1,+1], and consequently, the maximum likelihood method (Gath and Hayes, 2006) was adopted to estimate the parameters for the SEM analysis. The software used for all analyses was Amos 23.0 (Arbuckle, 2014).

## Results

Table 1 provides a summary of the main descriptive statistics for the variables under study (e.g. mean values and standard deviations), along with their zero-order correlations.

**Table 1:** Zero-order correlations and main descriptive statistics for mental health, life satisfaction and positive/negative school relations.

	1	2	3	4	5	6	7	8	9	10	11	12	13	14
1. Emotional MH	–													
2. Social MH	0.574^**^	–												
3. Psychological MH	0.716^**^	0.606^**^	–											
4. Sense of Community	0.366^**^	0.380^**^	0.393^**^	–										
5. Affiliation	0.330^**^	0.311^**^	0.344^**^	0.557^**^	–									
6. Bonds	0.362^**^	0.337^**^	0.382^**^	0.579^**^	0.690^**^	–								
7. Dissatisfaction	−0.279^**^	−0.238^**^	−0.281^**^	−0.363^**^	−0.568^**^	−0.421^**^	–							
8. Dangerousness	−0.172^**^	−0.105^**^	−0.188^**^	−0.322^**^	−0.292^**^	−0.360^**^	0.333^**^	–						
9. MSLSS family	0.416^**^	0.363^**^	0.396^**^	0.287^**^	0.298^**^	0.288^**^	−0.198^**^	−0.129^**^	–					
10. MSLSS friend	0.389^**^	0.379^**^	0.465^**^	0.423^**^	0.202^**^	0.242^**^	−0.128^**^	−0.186^**^	0.301^**^	–				
11. MSLSS environment	0.436^**^	0.467^**^	0.437^**^	0.350^**^	0.287^**^	0.297^**^	−0.195^**^	−0.120^**^	0.488^**^	0.395^**^	–			
12. MSLSS self	0.532^**^	0.478^**^	0.622^**^	0.317^**^	0.215^**^	0.271^**^	−0.194^**^	−0.135^**^	0.359^**^	0.518^**^	0.451^**^	–		
13. Age	−0.157^**^	−0.227^**^	−0.138^**^	−0.111^**^	−0.059^**^	−0.016	0.137^**^	−0.015	−0.098^**^	−0.064^**^	−0.187^**^	−0.209^**^	–	
14. Gender	−0.058^**^	−0.123^**^	0.003	−0.127^**^	−0.104^**^	−0.208^**^	0.056^**^	−0.048^**^	−0.064^**^	−0.005	−0.132^**^	0.056^**^	−0.018	–
*M*	13.12	15.79	26.23	21.91	21.88	23.01	6.38	4.60	20.67	20.15	13.96	20.90	16.69	
*SD*	3.27	5.72	6.35	3.87	4.52	3.70	1.90	1.59	5.00	3.28	3.40	3.66	1.45	
														

*M = mean; SD = standard deviation; sample size = 3,895; and reference group (male) = 1*.

** *p < 0.001.*

In general, the zero-order correlations revealed statistically significant, robustly positive patterns of association between mental health and positive school relations, with *r* values ranging between 0.382 (psychological mental health and bonds) and 0.311 (social mental health and affiliation). Furthermore, mental health was negatively associated with negative relations, with an especially robust association between dissatisfaction and the psychological dimension of mental health. However, the strongest patterns of association in terms of statistical significance and magnitude of effect were observed between mental health and life satisfaction, with values ranging between 0.622 (psychological mental health and satisfaction with self) and 0.379 (social mental health and satisfaction with friends). With regard to demographic variables, age was found to be negatively associated with all aspects of mental health and life satisfaction, while mixed patterns of association were found between age and school relations. Concerning gender differences, zero-order correlations were generally low or not statistically significant, with the exception of bonds with school (*r* = −0.108), a measure on which boys scored more poorly than girls. Overall, the correlational analysis provided support for testing a structural equation model with all the study variables.

The fit analysis (see Figure 1) suggested that the empirical data provided a good fit for conceptual model. All the fit indexes endorsed the full acceptance of the model: NFI = 0.954, NNFI = 0.954, CFI = 0.956, RMSEA = 0.066 [C.I. 90th = 0.063–0.070] and SRMR = 0.041. Total, direct and indirect standardised effects are reported in Table 2. Indirect effects reflect interaction among three variables, while a direct effect represents the impact of a single determinant on a given target variable.

**Table 2:** Breakdown of total, direct and indirect standardised effects identified *via* the structural model.

		Total effect			Direct effect			Indirect effect		
From	To	*β*	C.I. 90 [LB−UB]	*p*	*β*	C.I. 90 [LB−UB]	*p*	*β*	C.I. 90 [LB−UB]	*p*
Positive relations	Life satisfaction	0.465	[0.301–0.408]	0.015	0.465	[0.301–0.408]	0.015	–	–	–
Negative relations	Life satisfaction	−0.088	[−0.532–−0.058]	0.048	−0.088	[−0.532–-−.058]	0.048	–	–	–
Positive relations	Mental health	0.435	[0.270–0.371]	0.012	0.042	[−0.025–0.074]	0.388	0.393	[0.235–0.340]	0.011
Negative relations	Mental health	−0.151	[−0.770–−0.264]	0.007	−0.077	[−0.464–−0.084]	0.016	−0.074	[−0.431–−0.050]	0.045
Life satisfaction	Mental health	0.845	[0.762–0.869]	0.009	0.845	[0.762–0.869]	0.009	–	–	–
Age	Positive relations	−0.191	[−0.530–−0.396]	0.012	−0.191	[−0.530–−0.396]	0.012	–	–	–
Age	Negative relations	0.053	[0.011–0.053]	0.010	0.053	[0.011–0.053]	0.010	–	–	–
Age	Life satisfaction	−0.023	[−0.118–0.022]	0.274	0.071	[0.072–0.193]	0.009	−0.093	[−0.210–−0.141]	0.012
Age	Mental health	−0.050	[−0.154–−0.046]	0.010	−0.018	[−0.077–0.008]	0.177	−0.032	[−0.110–0.006]	0.139

*CI, confidence interval; LB, lower bound; UB, upper bound.*

**Figure 2:**
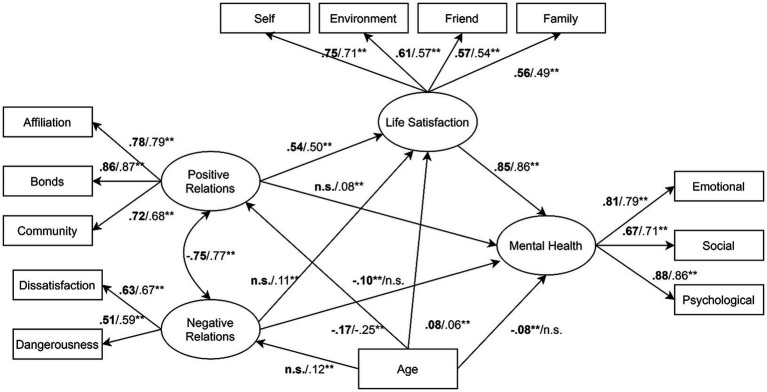
Structural model and standardised direct effects as resulting from invariance test (*n* = 3,895). ^**^p < 0.01; n.s. = not statistically significant. Values for males were reported in bold.

Again, moving from left to right, the latent variable, positive school relations, had significant effects on both life satisfaction (*β* = 0.478) and mental health (*β* = 0.435). Interestingly, the direct pathway between positive relations and mental health was negligible and non-significant (*β* = 0.042), meaning that life satisfaction fully mediated the association between these two variables (the magnitude of the indirect effect was 0.393). Negative school relations had significant total effects on both life satisfaction (*β* = − 0.088) and mental health (*β* = −0.151), although the magnitude of these effects was small compared to the effects of positive relationships. Finally, a large, statistically significant, positive pathway was observed from life satisfaction to mental health (*β* = 0.845). In terms of pathways between age and latent variables, older adolescents generally reported weaker positive relations (*β* = −0.191), poorer mental health (*β* = −0.05) and stronger negative relations (*β* = 0.053). In contrast, no statistically significant pathway was found between age and life satisfaction. The results of the invariance test of gender-based cohorts of adolescents are reported in Table 3 and Figure 2.

**Table 3:** Multigroup analysis of the structural equation model: fit indexes and model comparison.

Type	*χ*2 (df)	CFI	RMSEA	RMSEA 90% C.I.	Model comparison	Δ*χ*2 (Δdf)	Δ CFI	ΔRMSEA	Decision
M1. Configural invariance	947.2 (102)	0.956	0.047	[0.044–0.049]	–	–	–	–	Accept
M2. Metric invariance	998.3 (110)	0.954	0.046	[0.043–0.049]	M1	51.1 (8)	0.002	0.001	Accept
M3. Scalar invariance	1,365.3 (122)	0.935	0.052	[0.049–0.054]	M2	367.0 (12)	0.019	0.006	Reject
M4. Full invariance	1,569.4 (131)	0.925	0.059	[0.051–0.056]	M3	204.1 (9)	0.011	0.007	Reject

*df, degree of freedom; CFI, comparative fit index; RMSEA, root mean square error of approximation; CI, confidence interval.*

The main outcome of the invariance analysis was that the structural equation model supported the first two levels of invariance between boys and girls. Specifically, only configural and metric invariance were confirmed, meaning that the theorised set of variables and pathways held in the two groups, but males and females obtained different intercept values. Although the set of pathways remained substantially the same in the two cohorts of adolescents, there were minor differences concerning the latent variable negative relations: specifically, there was a small inverse association (*β* = −0.109) between negative relations and life satisfaction for girls, but this pathway was not statistically significant for boys.

## Discussion

The main aim of the present study was to investigate – *via* SEM – whether and to what extent quality of school relations and life satisfaction predicted mental health in a large sample of adolescents. We obtained the following key findings, which are discussed below in further detail. First, we found strong associations between the quality of adolescents’ school relations, their life satisfaction and their mental health. Second, life satisfaction, which was positively associated with mental health, was found to act as mediator between adolescents’ positive relationships and their mental health. Third, both the quality of school relations and life satisfaction appeared to protect mental health, and this outcome did not significantly vary as a function of gender. Finally, students’ quality of school relations and mental health deteriorated with age.

### Associations Among Quality of School Relations, Life Satisfaction and Mental Health

The first main outcome confirmed our leading research hypothesis, namely that positive school relations, in terms of affiliation with teachers, bond with school and sense of community, would be positively associated with mental health and life satisfaction. The second hypothesis was also borne out by the data: specifically, negative school relations, in terms of dissatisfaction with teachers and perceptions of school dangerousness, were negatively associated with life satisfaction and mental health. The structural equational model also indicated that the quality of school relations – operationalised *via* affiliation with teachers, bonds with school and sense of community – was associated with adolescents’ mental health. Although the literature acknowledges the importance of students’ social and relational experience, and especially the role of teachers in promoting it (e.g. Pianta, 1999; Roorda et al., 2011; Cavioni et al., 2017), few recent studies have been focused on how these factors may be related to one another during adolescence. Our findings also underscored the key role of student-teacher relationships, especially in the high school setting where they tend to become more impersonal (Roorda et al., 2011). This is in line with recent work by Ibrahim and El Zaatari (2020), who concluded that teacher-student relationships are the most important relationship in the school context and have positive effects on students’ mental health when characterised by empathy, closeness, love, care, support, respect and reciprocity. Another remarkable outcome of our model concerns the impact of adolescents’ self-perceived bonds with their school and sense of community at school on mental health. Although existing studies have examined the association between adolescents’ school bonds and sense of community on mental health, traditionally the focus has been on their role in reducing emotional and conduct issues, hyperactivity, peer problems, depression and anxiety (Lester et al., 2013; Gaete et al., 2016). Our findings, on the other hand, offer the novel insight that students’ school bonds and sense of community actually act as preventive factors. Consequently, mental health in schools needs to be promoted by building collective beliefs, values and expectations among students, teachers and all members of the school community. Overall, these outcomes are consistent with the positive psychology approach, which emphasises the importance of identifying and fostering positive indicators of mental health rather than merely detecting and seeking to mitigate factors that cause psychopathology (Seligman and Csikszentmihalyi, 2000; Antaramian et al., 2008).

### The Moderating Role of Life Satisfaction

Our second finding was that life satisfaction played a key mediating role between adolescents’ positive relationships at school and their mental health. In our model, life satisfaction was operationalised as adolescents’ subjective cognitive appraisal of their quality of life in the domains of self, environment, friends and family. We set out to investigate how contextual factors at school interact with individual characteristics to shape adolescents’ mental health, and our results suggest that life satisfaction, conceptualised as an individual factor, may play a significant part in this process. In addition, although the association between life satisfaction and mental health has been widely examined in adult populations, few studies have explored this aspect among adolescents. Existing research conducted with teenage samples has been focused on life satisfaction as a buffer against negative life events and stress, and the development of externalising and internalising behavioural problems (McKnight et al., 2002; Suldo and Huebner, 2004; Sawatzky et al., 2010). Our results suggest that life satisfaction for adolescents not only acts as a barrier mitigating the impact of negative events and mental health problems (Lazarus, 1991; Suldo and Huebner, 2004) but also represents a key psychological resource through which positive relationships can boost mental health.

### Gender Invariance

Overall, we identified minor gender differences in the relative strength of the associations among the variables. Previous studies with adolescents mainly examined the role of gender in terms of differential patterns of internalising and externalising behavioural problems in boys versus girls (e.g. van der Ende and Verhulst, 2005). However, in this study, we explored the role of gender by applying a multigroup invariance test to compare the performance of our conceptual model across gender groups (Ornaghi et al., 2016). Our structural equation model was aimed at identifying contextual and individual resources that contribute to mental health rather than comparing psychological symptoms between genders. The results suggested that positive school relations and life satisfaction protect the school mental health of both girls and boys.

### Age Invariance

We found that older students reported weaker positive school relations and poorer mental health, along with a higher incidence of negative school relations. These findings are consistent with previous studies that have identified declines over time in adolescents’ mental health outcomes as well as in their perceptions of emotional support from teachers (Eccles and Roeser, 2011; Bayram Özdemir and Özdemir, 2020). Given that adolescence is a major life period marked by numerous individual and contextual challenges with the potential to impact on adolescents’ mental health (Dwivedi and Harper, 2004; Grazzani Gavazzi et al., 2011), our results add to our understanding of the association between school relations and mental health across age groups, confirming that student mental health hits its lowest point during late adolescence.

### Limitation and Future Studies

Some limitations of this study should be noted. First, although we analysed data from a large sample of adolescents, the outcomes cannot be generalised to early adolescence nor to adolescents with atypical development. Second, the study was conducted on a sample of adolescents mainly enrolled at lyceums. Consequently, the findings can be generalised with caution to the whole population of high school students attending professional and technical institutes. Third, given that the present data were collected in Italian schools from only two regions, the outcome could not be automatically extended to adolescents from the whole Italian Country.

Finally, while we examined the impact of school relations and life satisfaction on adolescents’ mental health at a single time point, relationships at school are never static. We, therefore, recommend that the future research adopt a longitudinal design to investigate how the contribution of the school relationships to young people’s mental health may evolve over time.

## Data Availability Statement

The raw data supporting the conclusions of this article will be made available by the authors, without undue reservation.

## Ethics Statement

The studies involving human participants were reviewed and approved by the University of Milano-Bicocca. Written informed consent to participate in this study was provided by the participants’ legal guardian/next of kin.

## Author Contributions

VC has made substantial contributions to the conception and design of the research, to the collection, input, scoring and interpretation of the data, and drafting of the manuscript. IG made a key contribution to designing the research, interpreting the data, drafting and revising the manuscript. VO contributed to interpreting the data and revising the manuscript critically. AA has been involved in collecting the data and revising the manuscript critically. AP made a key contribution to analysing and interpreting the data and in drafting the manuscript. All authors read and approved the final manuscript.

## Conflict of Interest

The authors declare that the research was conducted in the absence of any commercial or financial relationships that could be construed as a potential conflict of interest.

## Publisher’s Note

All claims expressed in this article are solely those of the authors and do not necessarily represent those of their affiliated organizations, or those of the publisher, the editors and the reviewers. Any product that may be evaluated in this article, or claim that may be made by its manufacturer, is not guaranteed or endorsed by the publisher.
